# Supplementary figures and images for: Tribbles1 is host protective during in vivo mycobacterial infection
Source: eLife. 2024 Jun 18;13:e95980. doi: 10.7554/eLife.95980 (PMC11186633; doi:10.7554/eLife.95980)

A

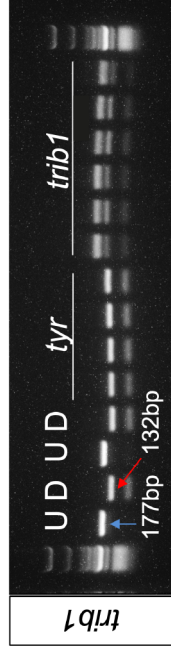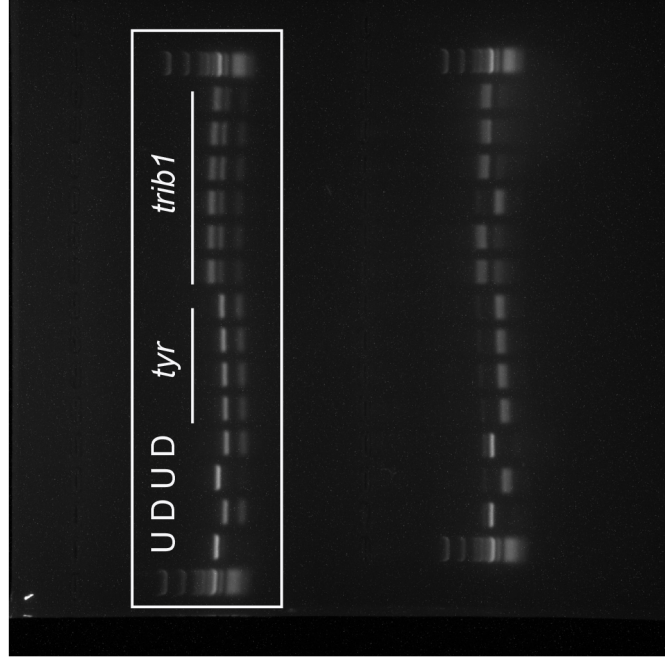

Supplement: Figure 4—figure supplement 2—source data 2. [file elife-95980-fig4-figsupp2-data2.pdf]

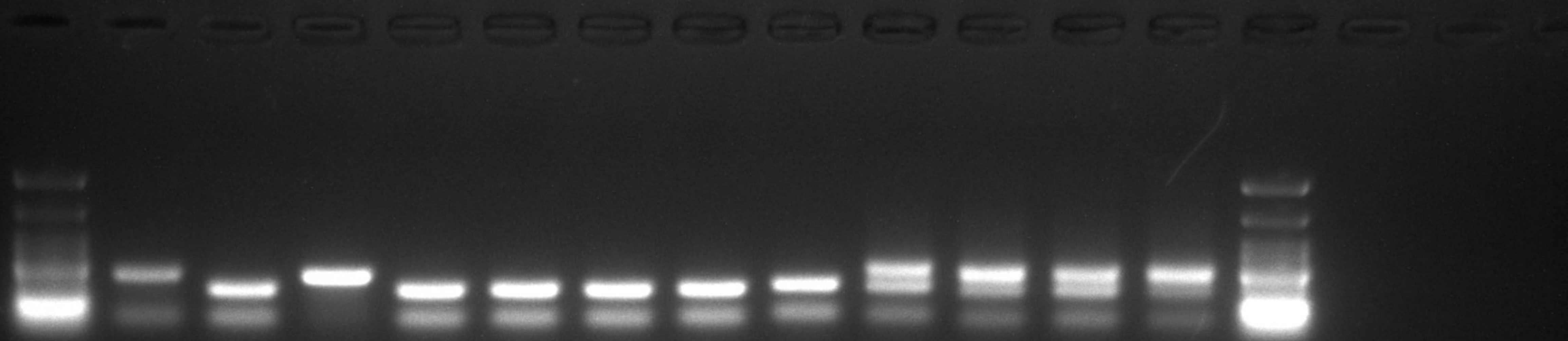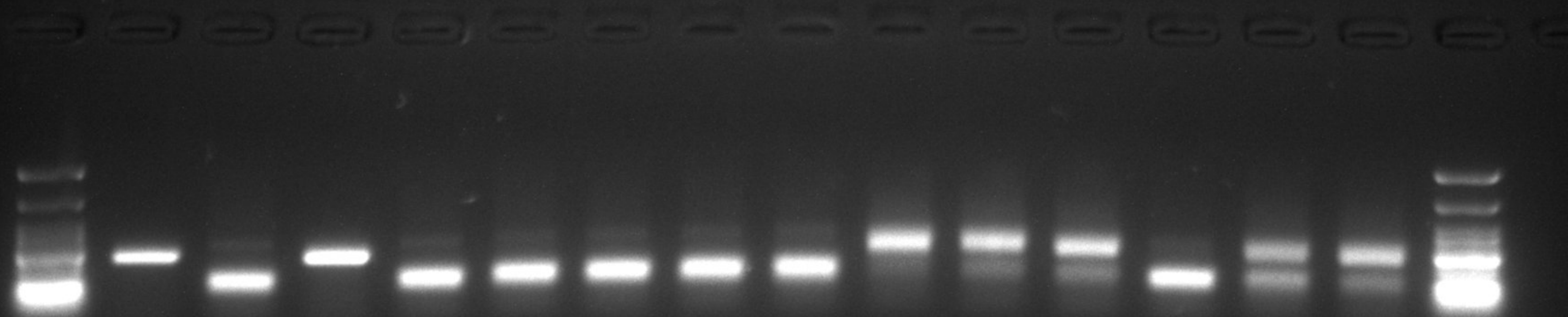

Supplement: Figure 4—figure supplement 2—source data 3. [file elife-95980-fig4-figsupp2-data3.pdf]

B

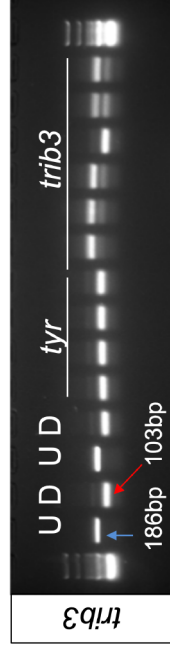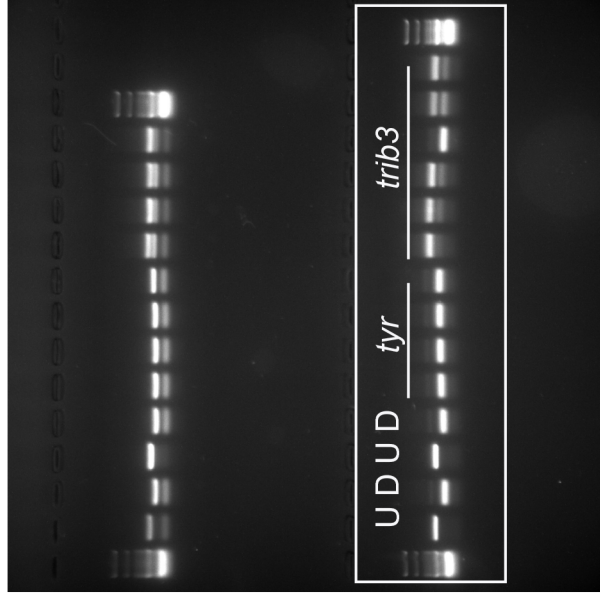

Supplement: Figure 4—figure supplement 2—source data 4. [file elife-95980-fig4-figsupp2-data4.pdf]

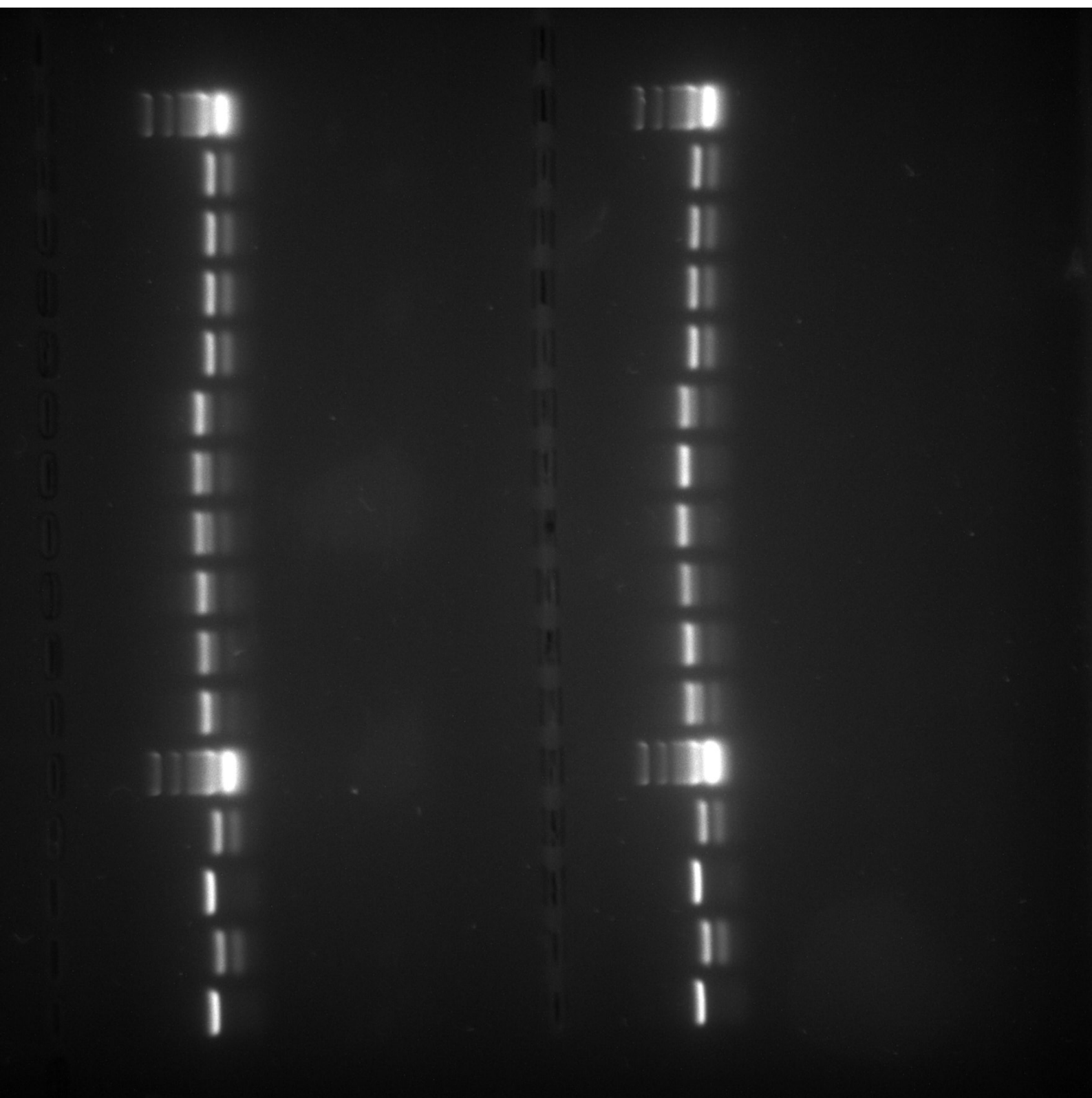

Supplement: Figure 4—figure supplement 3—source data 1. [file elife-95980-fig4-figsupp3-data1.pdf]

B

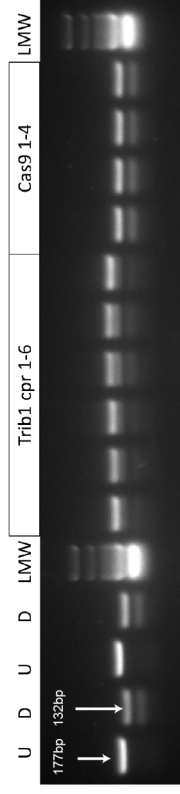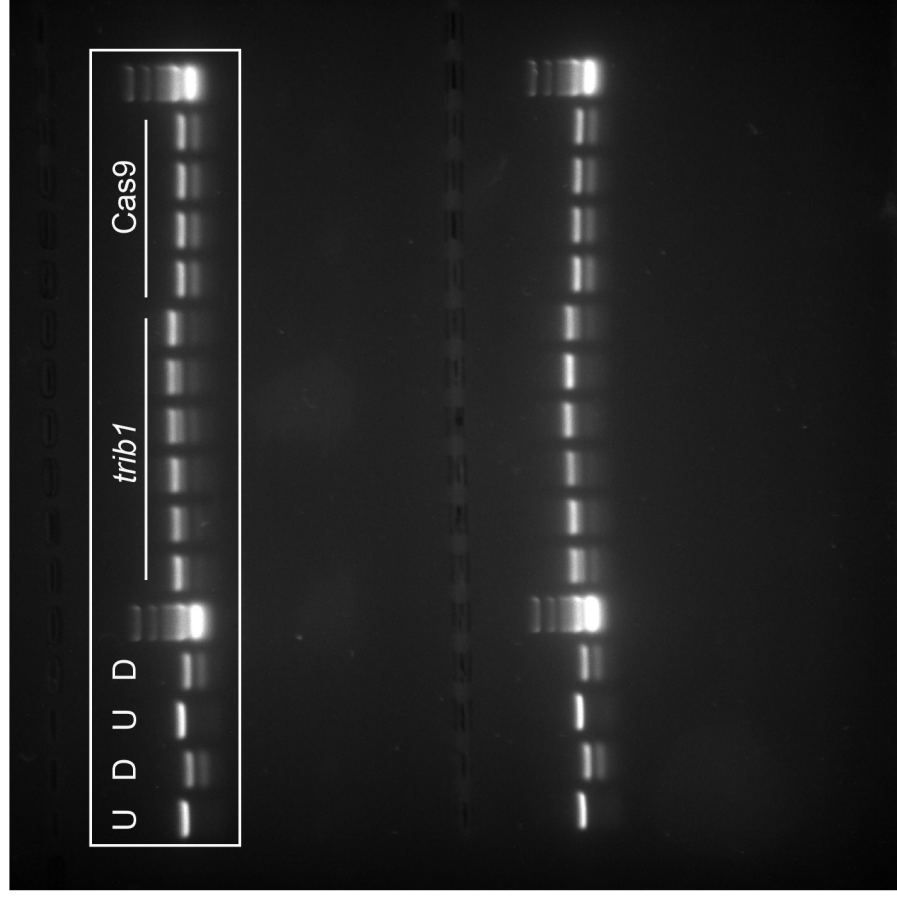

Supplement: Figure 4—figure supplement 3—source data 2. [file elife-95980-fig4-figsupp3-data2.pdf]

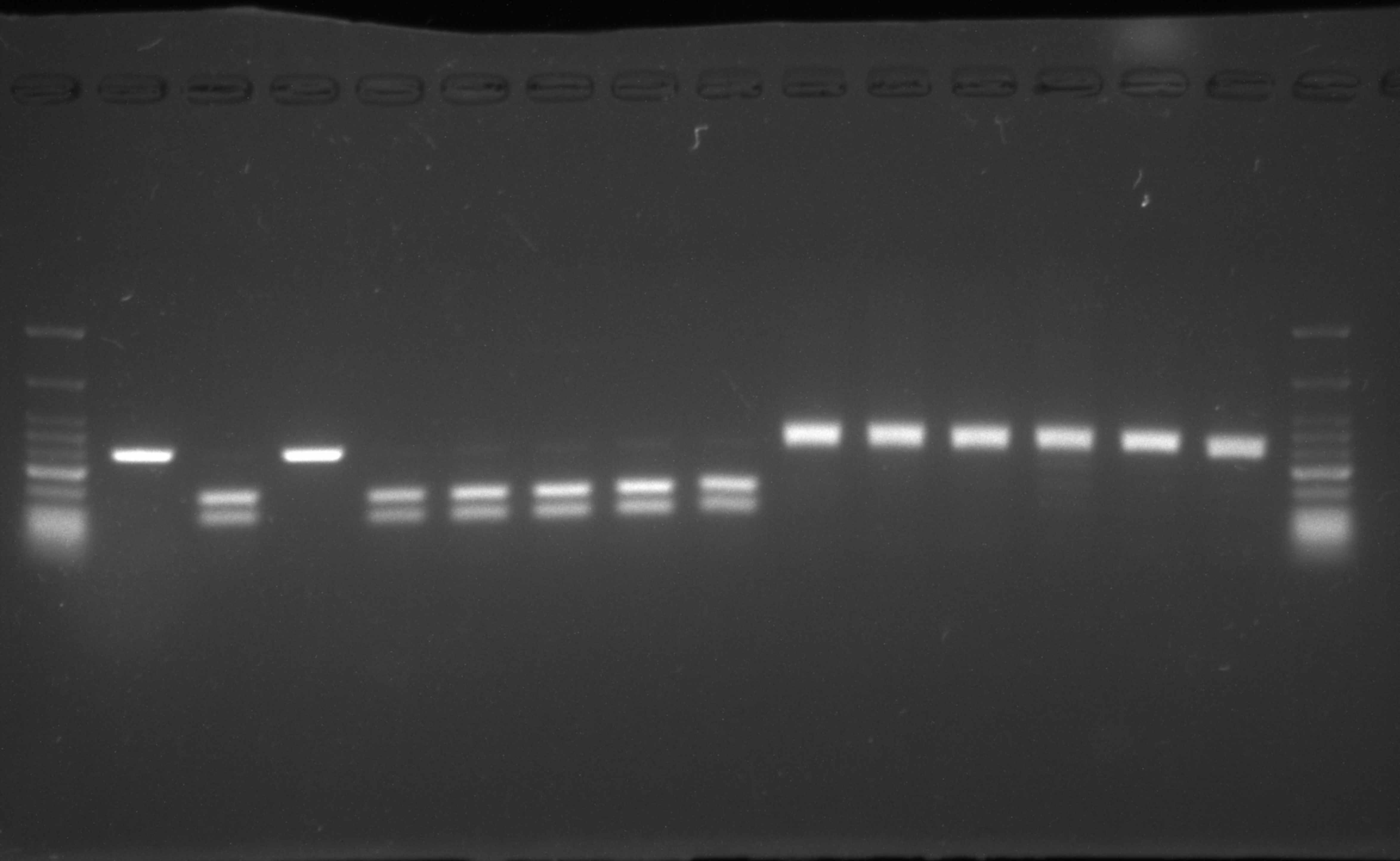

Supplement: Figure 8—figure supplement 1—source data 1. [file elife-95980-fig8-figsupp1-data1.pdf]

B

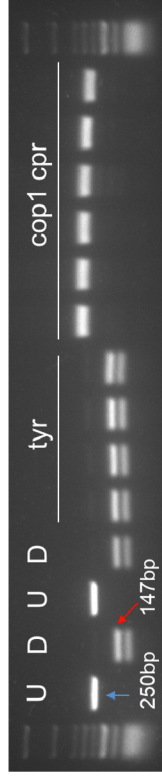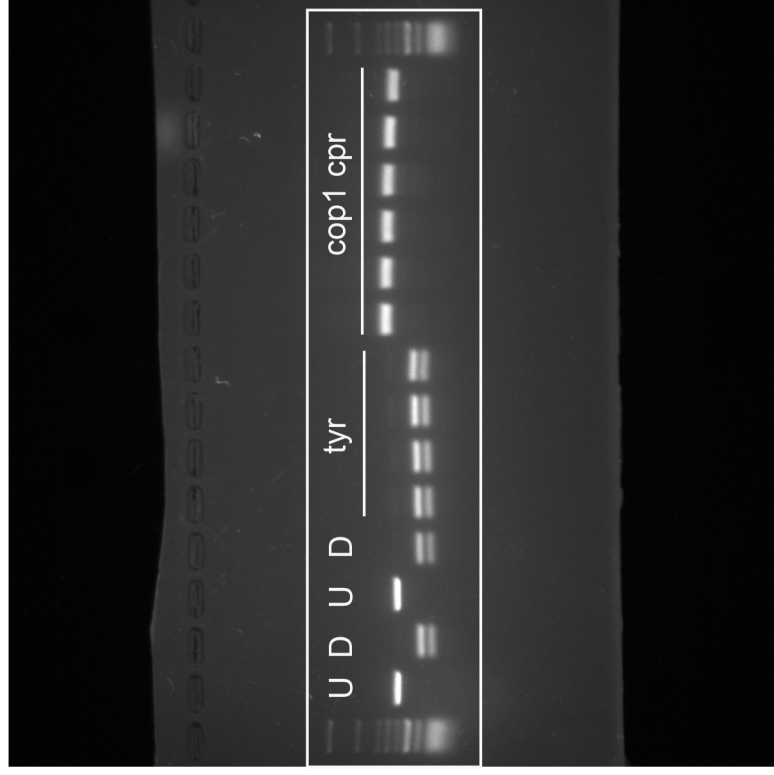

Supplement: Figure 8—figure supplement 1—source data 2. [file elife-95980-fig8-figsupp1-data2.pdf]
