# Supplementary material for: Tribbles1 is host protective during in vivo mycobacterial infection
Source: eLife. 2024 Jun 18;13:e95980. doi: 10.7554/eLife.95980 (PMC11186633; doi:10.7554/eLife.95980)

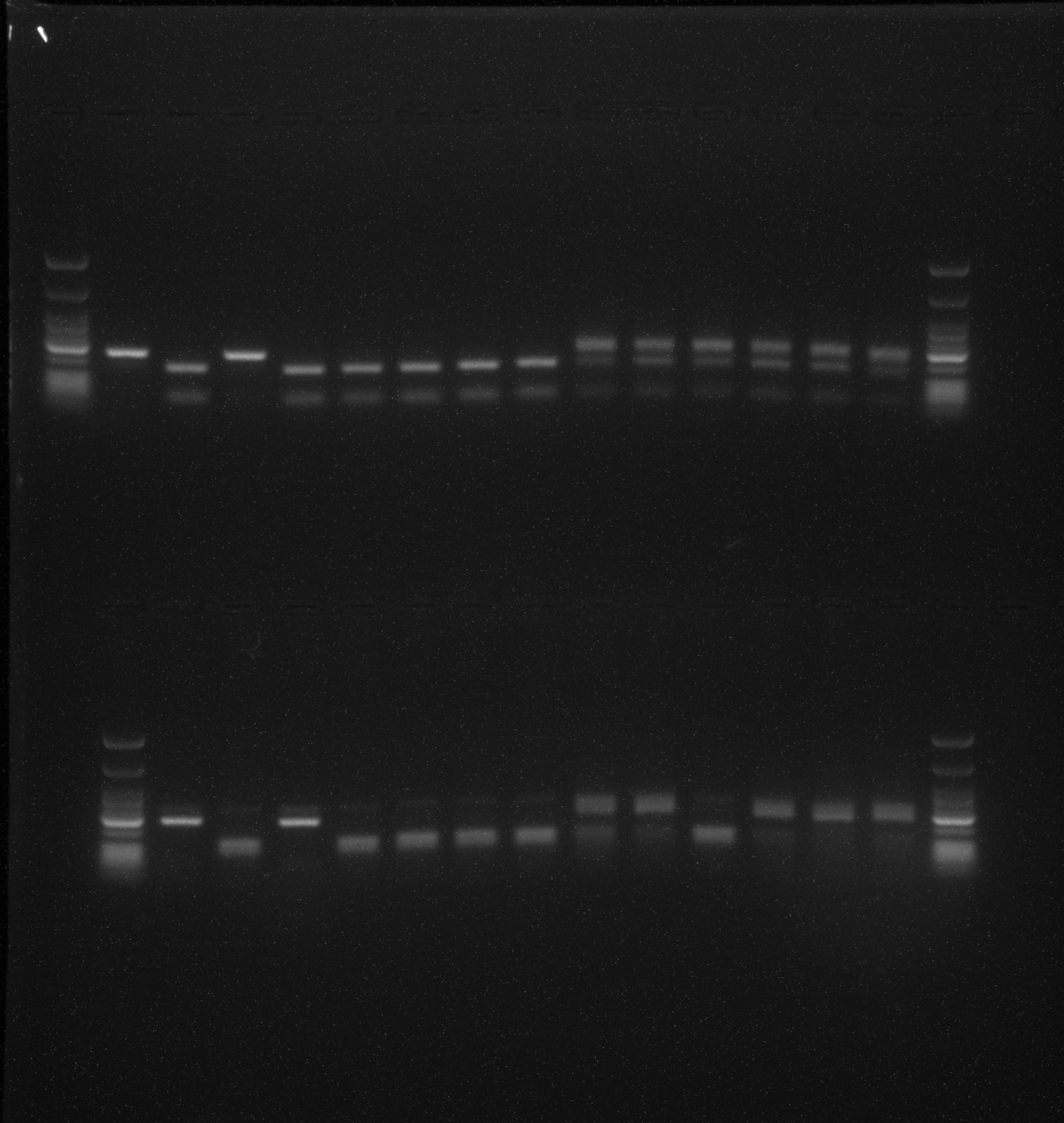

Supplement: Figure 4—figure supplement 2—source data 1. [file elife-95980-fig4-figsupp2-data1.pdf]
